# Comparison of Multiple Diagnostic Tests to Measure Dynamic Hyperinflation in Patients with Severe Emphysema Treated with Endobronchial Coils

**DOI:** 10.1007/s00408-021-00430-0

**Published:** 2021-03-09

**Authors:** Marlies van Dijk, Jorine E. Hartman, Sonja W. S. Augustijn, Nick H. T. ten Hacken, Karin Klooster, Dirk-Jan Slebos

**Affiliations:** Department of Pulmonary Diseases, AA11, University Medical Center Groningen, PO Box 30001, 9700 RB Groningen, The Netherlands

**Keywords:** COPD, Emphysema, Dynamic hyperinflation, Endobronchial coils

## Abstract

**Purpose:**

For this study, we aimed to compare dynamic hyperinflation measured by cardiopulmonary exercise testing (CPET), a six-minute walking test (6-MWT), and a manually paced tachypnea test (MPT) in patients with severe emphysema who were treated with endobronchial coils. Additionally, we investigated whether dynamic hyperinflation changed after treatment with endobronchial coils.

**Methods:**

Dynamic hyperinflation was measured with CPET, 6-MWT, and an MPT in 29 patients before and after coil treatment.

**Results:**

There was no significant change in dynamic hyperinflation after treatment with coils. Comparison of CPET and MPT showed a strong association (rho 0.660, p < 0.001) and a moderate agreement (BA-plot, 202 ml difference in favor of MPT). There was only a moderate association of the 6-MWT with CPET (rho 0.361, p 0.024).

**Conclusion:**

MPT can be a suitable alternative to CPET to measure dynamic hyperinflation in severe emphysema but may overestimate dynamic hyperinflation possibly due to a higher breathing frequency.

## Introduction

In patients with emphysema, reduced elastic recoil of the lungs and increased airway resistance lead to static hyperinflation, i.e. an increased end expiratory lung volume. During exercise, due to a higher breathing frequency, the end expiratory lung volume may further increase at the expense of the inspiratory capacity (IC). This so-called dynamic hyperinflation contributes to dyspnea, reduced exercise tolerance and reduced quality of life [1].

There are various techniques to measure dynamic hyperinflation. Measuring the reduction in IC during cardiopulmonary exercise testing (CPET) is most commonly used and considered to be the reference standard [2]. Reduction in IC can also be measured with a metronome or manually paced tachypnea test (MPT), where the patient is instructed to breath with a high frequency for a set amount of time, or with a six-minute walk test (6-MWT) [3, 4].

Bronchoscopic lung volume reduction with endobronchial coils is a guideline treatment for selected patients with severe static hyperinflation and emphysema [5]. Coil treatment can lead to an improvement in static hyperinflation, exercise tolerance, and health status [6, 7].

For this study, we aimed to compare dynamic hyperinflation measured by CPET, 6-MWT, and MPT in patients with very severe emphysema who were treated with endobronchial coils. More specifically, we wanted to assess the accuracy and agreement of measuring dynamic hyperinflation with the MPT and 6-MWT compared to CPET. Additionally, we investigated whether dynamic hyperinflation changed after treatment with endobronchial coils.

## Methods

This single-center prospective study included patients with severe emphysema and hyperinflation who participated in an open-label trial investigating endobronchial coil treatment which was approved by the local ethics committee (NCT02179125). All patients provided written informed consent. Patients were included between September 2015 and November 2017.

The coil treatment was performed during two separate bronchoscopic procedures under fluoroscopy while the patient was under general anesthesia. During each bronchoscopy, 9 to 13 nitinol coils were placed in one (usually upper) lobe. After 6 weeks, the second target lobe was treated during a second bronchoscopy, unless there were complications of the first treatment, in which case the second procedure was postponed or canceled.

All measurements were performed at baseline and three months post-treatment. Post-bronchodilator spirometry, body plethysmography, and diffusing capacity were measured according to ATS/ERS guidelines [8, 9]. The CPET (an incremental cycle-ergometer test) and 6-MWT were performed according to current guidelines [2, 10]. For both tests, IC was measured with the use of a pneumotachograph beforehand (the mean of three reproducible measurements, defined as < 150 ml and/or < 5% between measurements) and 30 s after maximal exercise (mean of two measurements). IC was measured in a semi-recumbent position for the CPET and standing upright for the 6-MWT. The protocol for measuring dynamic hyperinflation with MPT was described in an earlier publication [3]. In short, after measurement of the baseline IC, patients were asked to breathe 40 times/minute for one minute, after which the IC was immediately measured again. This measurement was repeated three times. Dynamic hyperinflation was calculated by subtracting the IC post-test from the IC pre-test.

## Results

Twenty nine patients were included: 21 female; median age 63 (range 44 to 76) years; FEV_1_ 25 (14 to 43)%pred; and RV 231 (176 to 322)%pred. Treatment with coils resulted in a change in FEV_1_ of + 95 (− 5 to + 320) ml, RV -390 (− 1490 to + 10) ml, and six-minute walking distance + 28 (-7 to + 174) meters (all p ≤ 0.001, Wilcoxon Signed Rank test). At the baseline CPET, the median workload was 27 (3 to 51) Watt, with a peak VO_2_ of 10.0 (7.7 to 15.4) ml/min/kg. In post-treatment, there was a clinically relevant change in workload of + 7 (− 5 to + 16) Watt (p < 0.001) [11], with no significant change in peak VO_2_ (+ 0.2 (− 2.2 to + 2.9)).

At baseline, dynamic hyperinflation was − 740 (− 1530 to − 170) ml, − 900 (− 1470 to − 540) ml and − 335 (− 690 to + 60) ml measured with CPET, MPT, and 6-MWT, respectively (Table 1). These differences between baseline dynamic hyperinflation per test were statistically significant (all *p* < 0.01). Three months after treatment, there was no significant change in dynamic hyperinflation measured with CPET (0 (− 750 to + 430)ml, *p* 0.93), MPT (− 18 (− 550 to + 230)ml, *p* 0.17), and 6-MWT (− 85 (− 510 to + 500) ml, *p* 0.11). In post-treatment, there was a significant change in tidal volumes during maximum effort for CPET (+ 110 (− 10 to + 710) ml, *p* 0.001) and average tidal volumes for MPT (+ 55 (− 55 to + 260) ml, *p* < 0.001) (Table 1).

**Table 1:** Baseline and follow-up outcomes for manually paced tachypnea test (MPT), cardiopulmonary exercise test (CPET), and six-minute walk test (6-MWT)

	Baseline	Follow up	p value
CPET			
Dynamic hyperinflation (ml)	− 740 (− 1530 to − 170)	− 750 (− 1300 to − 320)	0.93
Breathing frequency (x/min) at maximum workload	28 (16–55)	26 (15–38)	0.017
Tidal volumes (ml) at maximum workload	840 (480–1310)	1010 (630–1780)	0.001
Maximum workload (Watt)	27 (3–51)	33 (16–61)	0.001
VO_2_ peak (ml/min)	719 (508–1090)	770 (546–1109)	0.13
MPT			
Dynamic hyperinflation (ml)	− 900 (− 1470 to − 540)	− 990 (− 1690 to − 540)	0.017
Breathing frequency (x/min)	40 (39–42)	40 (39–41)	0.53
Tidal volumes (ml)	589 (390–895)	680 (460–975)	< 0.001
6-MWT			
Dynamic hyperinflation (ml)	− 335 (− 690 to + 60)	− 480 (− 930 to − 110)	0.11
Distance	321 (172–469)	362 (160–469)	0.004

VO_2_ = oxygen uptake

**Fig. 1:**
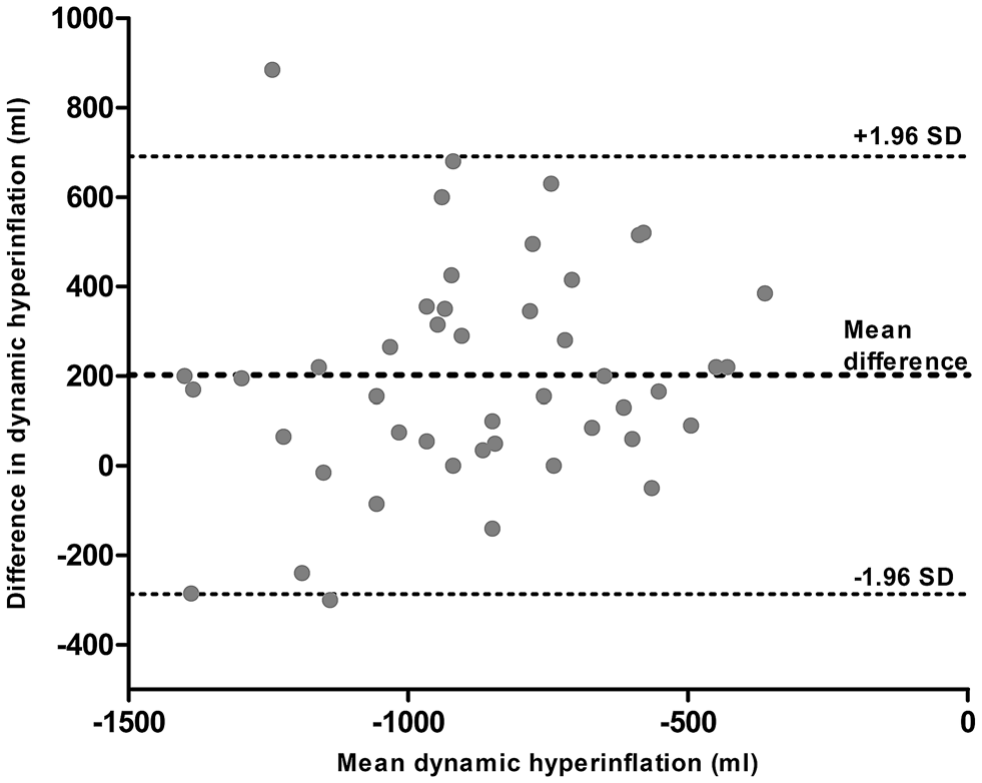
A Bland–Altman plot of dynamic hyperinflation measured by cardiopulmonary exercise testing (CPET) and a manually paced tachypnea test (MPT)

There was a strong association between dynamic hyperinflation measured by CPET and MPT (rho 0.660, *p* < 0.001), and a moderate association between CPET and the 6-MWT (rho 0.361, *p* 0.024). Measurements of dynamic hyperinflation with CPET and MPT were plotted in a Bland–Altman plot (Fig. 1). There was a mean difference of 202 ml (95% CI − 287 to + 690 ml) between dynamic hyperinflation measured by CPET and MPT.

## Discussion

In this single-center prospective study, dynamic hyperinflation was measured in patients with severe emphysema using cardiopulmonary exercise testing, a manually paced tachypnea test, and a 6-min walk test. There was a strong significant association between dynamic hyperinflation measured by CPET and MPT. A Bland–Altman plot showed a moderate agreement between these two tests, with a mean difference of dynamic hyperinflation of 202 ml in favor of MPT. We found only a moderate association of 6-MWT with CPET.

We previously demonstrated measurement of dynamic hyperinflation with MPT to be safe and feasible in patients with severe COPD [3]. In addition, this study demonstrates a strong association and moderate agreement between dynamic hyperinflation measurements with CPET and MPT. This strong association is in line with earlier studies [12, 13]. An advantage of MPT over CPET is that it is less costly and time consuming.

A possible explanation for the 202 ml mean difference between dynamic hyperinflation measured with CPET and MPT in our study could be the difference in maximum breathing frequency (CPET: 26/min at maximum workload versus MPT: 40/min). MPT may, therefore, overestimate dynamic hyperinflation, since in real life, patients with severe emphysema may rarely reach a breathing frequency this high. Other possible explanations are a different interval between the end of the test and the IC measurement, a different posture (CPET: semi-recumbent versus MPT: sitting), and a different exercise state. However, earlier studies in patients with moderate to severe emphysema did not find a significant difference in dynamic hyperinflation between MPT and CPET [12, 13].

In this study, the 6-MWT appears to be a less suitable alternative for CPET to measure dynamic hyperinflation. During the study, the interval to IC measurement at the end of the test was often over the prespecified 30 s, mainly because of exhaustion of the patient who was then unable to correctly perform the IC maneuver after this short interval. This may explain in part why dynamic hyperinflation measured with the 6-MWT was significantly lower than CPET and MPT.

There was no difference between dynamic hyperinflation before and after coil treatment in the current study. This is different from an earlier study we performed demonstrating an increase in dynamic hyperinflation measured by MPT after bronchoscopic lung volume reduction with either coils or endobronchial valves [14]. The proposed underlying mechanism for this increase in dynamic hyperinflation is an increase in the inspiratory capacity because of the reduction in static hyperinflation, which leaves more room for dynamic hyperinflation to occur. In the current study, the improvement in static hyperinflation was less pronounced than in the earlier study (-390 versus -765 ml), which may in part explain why dynamic hyperinflation did not change. Furthermore, for the CPET and 6-MWT, there was a median increase in exercise capacity after treatment (+ 7 W and + 28 m, respectively), which means that the same amount of dynamic hyperinflation occurred at a later moment during exercise. However, this does not apply to the MPT, which is based on hyperventilation without exercise.

In the current study, all patients had severe static hyperinflation and airflow obstruction and demonstrated an impaired exercise capacity on the basis of a ventilatory limitation during CPET. Both at baseline and follow-up, dynamic hyperinflation was demonstrated in all participating subjects. So even after coil treatment, dynamic hyperinflation is still likely to contribute to the reduced exercise tolerance. Therefore, one could argue that measuring the presence of dynamic hyperinflation in this patient category may not give additional information, since it is already highly likely that dynamic hyperinflation is present. However, to learn more about the quantity of dynamic hyperinflation and the effects of new treatments on dynamic hyperinflation, we believe it is still useful to perform measurements of dynamic hyperinflation in these patients.

Study limitations are the relatively small sample size and that we investigated a selected group of emphysema patients with severe static hyperinflation, since these are prerequisites for bronchoscopic lung volume reduction treatments. Furthermore, there were some differences in the interval between the end of exercise or hyperventilation and the moment, the IC was measured between the three investigated tests, which could account for some of the differences in measured dynamic hyperinflation.

In conclusion, we demonstrated that a manually paced tachypnea test is a suitable alternative to cardiopulmonary exercise testing to measure dynamic hyperinflation in patients with severe emphysema, although it may slightly overestimate dynamic hyperinflation. The 6-min walking test was a less suitable substitute to cardiopulmonary exercise testing for the measurement of dynamic hyperinflation. Furthermore, we measured no change in dynamic hyperinflation after treatment with endobronchial coils.

## Data Availability

The datasets used and/or analyzed during the current study are available from the corresponding author on reasonable request.
